# Supplementary material for: How do authors’ perceptions of their papers compare with co-authors’ perceptions and peer-review decisions?
Source: PLoS One. 2024 Apr 10;19(4):e0300710. doi: 10.1371/journal.pone.0300710 (PMC11006147; doi:10.1371/journal.pone.0300710)
Supplement: Attachment — Submitted filename: response to reviewers.pdf [file pone.0300710.s001.pdf]

RESPONSE LETTER

Note from authors: In this document, we respond to questions from the editor and the reviewers. In this letter, we reproduce the text from the editor's and reviewers' responses in **black** and provide our corresponding response in **blue.**

Thank you to the Academic Editor and the reviewers for their suggestions and positive feedback about our work! We have incorporated your suggestions in our manuscript. We provide a detailed response to your comments below.

>> Reviewer #1: This paper confirms empirically what has been anecdotally known, that authors have a much higher expectation for acceptance of their work, even for venues where acceptance rates are very low. I applaud the authors of this paper for confirming this at a very large scale. As the authors note, there could be selection bias, and it is difficult to tell whether authors who simply did not opt into the survey had higher or lower expectations of acceptance.

It is interesting to note that authors who were invited to be meta reviewers and reviewers were better calibrated. This is also not a surprising result, as these are senior authors who are both experienced and also less invested in each submission (compared to more junior authors who were probably main authors rather than corresponding).

I am a bit concerned about the gender difference reported, as there could be intersectionality here. Are there more senior male authors than senior female authors? In that case, would it make sense to control for seniority?

Response: Thank you for your positive feedback, and your time and consideration in reviewing our submission. Regarding your comments on controlling for seniority in our gender-based analysis: We do indeed control for demographic factors, particularly, seniority and geographical region in our analysis, the specific details are provided in Appendix B.

Following up on your feedback, we have emphasised this detail of our analysis in the main text by adding the following text at the beginning of Section 5.2.1:

"We compute the average calibration error for gender subgroups using the gender data retrieved from participants' Open Review profiles. Our calculation accounts for potential confounding by seniority and geographic region in the analysis for calibration error across gender groups. Our approach is provided in Appendix B."

Another concern is about the data release. I understand that compromising reviewer confidentiality would be problematic, but it would be nice to share this dataset somehow with the community.

Response: In our work, we analyze data obtained from the scientific conference NeurIPS (Neural Information Processing Systems) 2021 for publication of research in ML. For this, we worked in collaboration with the conference organizers of NeurIPS 2021 (Alina Beygelzimer, Yann N. Dauphin, Percy Liang, Jennifer Wortman Vaughan, Zhenyu Xue) who are co-authors on this work. We are not able to share this data in any form, because of the following reasons:

1. This is highly sensitive data concerning authors and reviewers both, where authors and reviewers provide their honest opinion with the belief that this would not affect them personally.

2. NeurIPS 2021 is a double-blind conference and maintaining the double-blindness is an important aspect of the reviewing process. Releasing de-identified data would dilute the double-blindness.

3. It would violate the confidentiality agreement between the researchers who participated in the conference and the conference organizers. In the survey form used to collect data from participants, we clearly mentioned that:

"Only aggregate statistics will be released: Any information will be anonymized and only reported in an aggregated manner that protects your identities.

Data deletion: All the data pertaining to the survey (except for the aggregated results) will be entirely deleted by December 31, 2022."

Therefore, we are only able to share the aggregated statistics, which are provided in the manuscript, and are unable to share any other data collected.

Further, any other requests or questions may be sent to The Neural Information Processing Systems Foundations Board, the board responsible for running the conference NeurIPS 2021, or to the Institutional Review Board of Microsoft Research (contact via email to MSRethics@microsoft.com).

>> Reviewer #2: This is an interesting and well designed study. I have only a couple of minor comments.
1. Some additional proofreading is needed. Most of the verbs were stated in present tense when they should have been past tense.
2. The overall findings should be summarized in the conclusion, then the limitations should be discussed.
3. It might be useful to speculate about how the problem of overestimation of the likelihood of acceptance impacts the trust in peer review. Could it lead to dissatisfaction?

Response: Thank you for your positive feedback, and for your suggestions to improve the submission. We have incorporated your suggestions and provide a response for each, below:

1. Thank you for pointing this out, we have taken a pass over the entire document and fixed the tense as needed.

2. We have added a short summary to the conclusion, the text is reproduced here for your convenience:

   "This work highlights the perception gap in the peer-review process, between authors, co-authors, and reviewers. Through surveys conducted in NeurIPS 2021, we found that authors considerably overestimated the chances of their papers getting accepted. Our analysis also shed light on the difference in estimation error across demographic-based subgroups. Next, we considered differences in perception across reviewers and authors to find that authors disagreed with peer-review decisions roughly 30\% of the times, and co-authors disagreed with each other roughly 30\% of the times. Finally, more than 30\% of all survey respondents noted that their perception of their submission improved after the review process. "

3. You raise a good point. There is past literature on how "the culture of rejection" in academia leads to an overall bad experience for students (and more generally early career researchers). We added a discussion on this to the discussion section, the text is reproduced here:

   "Importantly, peer-review outcomes have been known to impact the graduate school experience of PhD students, with repeated rejections an oft-cited cause for worsening mental health [1]. We hope that the findings will help in cultivating awareness among the student research community about their perceptions as a whole, and facilitate discussions between students and advisors about setting appropriate expectations from the peer-review process, thereby fostering a healthier relationship with their work.."

[1] L. M. Jaremka et. al. "Common Academic Experiences No One Talks About: Repeated Rejection, Impostor Syndrome, and Burnout". In: Perspectives on Psychological Science, 15(3), 519–543 (2020).
